# (2*E*)-3-(2-Chloro­benzo[*h*]quinolin-3-yl)-1-(2-methyl-4-phenyl­quinolin-3-yl)prop-2-en-1-one

**DOI:** 10.1107/S1600536813021545

**Published:** 2013-08-07

**Authors:** R. Prasath, S. Sarveswari, Seik Weng Ng, Edward R. T. Tiekink

**Affiliations:** a Department of Chemistry, BITS, Pilani – K. K. Birla Goa Campus, Goa 403 726, India; b Centre for Organic and Medicinal Chemistry, School of Advanced Sciences, VIT University, Vellore 632 014, India; c Department of Chemistry, University of Malaya, 50603 Kuala Lumpur, Malaysia; d Chemistry Department, Faculty of Science, King Abdulaziz University, PO Box 80203 Jeddah, Saudi Arabia

## Abstract

In the title compound, C_32_H_21_ClN_2_O, an almost planar (r.m.s. deviation = 0.033 Å) prop-2-en-1-one bridge links quinolinyl and benzoquinolinyl residues; the latter are twisted out of the plane of the bridge [dihedral angles = 75.94 (5) and 20.20 (5)°, respectively]. In the crystal, a three-dimensional architecture arises as a result of C—H⋯O, C—H⋯π and π–π [centroid–centroid distances involving pyridine rings = 3.5806 (7)–3.7537 (7) Å] interactions.

## Related literature

For biological applications of quinoline derivatives, see: Jörg *et al.* (2007 ▶); Prasath *et al.* (2013*a* ▶). For a related structure, see: Prasath *et al.* (2013*b* ▶).
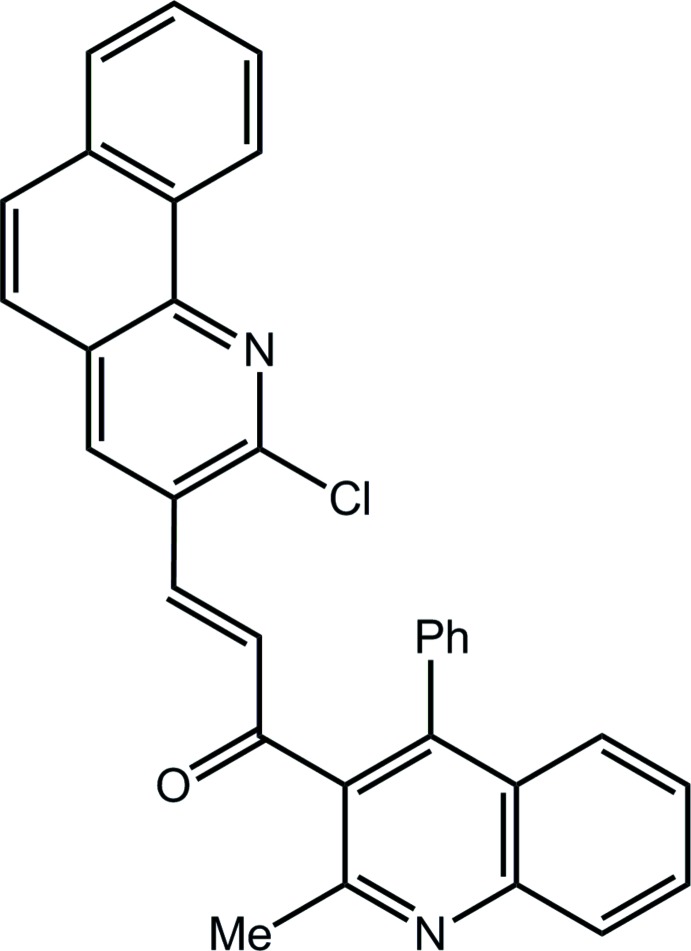


## Experimental

### 

#### Crystal data

C_32_H_21_ClN_2_O
*M* *_r_* = 484.96
Triclinic, 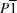

*a* = 7.1354 (3) Å
*b* = 10.1627 (5) Å
*c* = 17.0127 (8) Å
α = 78.758 (4)°
β = 79.544 (4)°
γ = 84.042 (4)°
*V* = 1186.91 (9) Å^3^
*Z* = 2
Cu *K*α radiation
μ = 1.65 mm^−1^
*T* = 100 K
0.30 × 0.25 × 0.20 mm

#### Data collection

Agilent SuperNova Dual diffractometer with an Atlas detector
Absorption correction: multi-scan (*CrysAlis PRO*; Agilent, 2013 ▶) *T*_min_ = 0.870, *T*_max_ = 1.000
8673 measured reflections
4849 independent reflections
4433 reflections with *I* > 2σ(*I*)
*R*_int_ = 0.020

#### Refinement

*R*[*F* ^2^ > 2σ(*F* ^2^)] = 0.034
*wR*(*F* ^2^) = 0.095
*S* = 1.04
4849 reflections
326 parameters
H-atom parameters constrained
Δρ_max_ = 0.24 e Å^−3^
Δρ_min_ = −0.31 e Å^−3^

### 

Data collection: *CrysAlis PRO* (Agilent, 2013 ▶); cell refinement: *CrysAlis PRO*; data reduction: *CrysAlis PRO*; program(s) used to solve structure: *SHELXS97* (Sheldrick, 2008 ▶); program(s) used to refine structure: *SHELXL97* (Sheldrick, 2008 ▶); molecular graphics: *ORTEP-3 for Windows* (Farrugia, 2012 ▶) and *DIAMOND* (Brandenburg, 2006 ▶); software used to prepare material for publication: *publCIF* (Westrip, 2010 ▶).

## Supplementary Material

Crystal structure: contains datablock(s) general, I. DOI: 10.1107/S1600536813021545/pv2642sup1.cif

Structure factors: contains datablock(s) I. DOI: 10.1107/S1600536813021545/pv2642Isup2.hkl

Supplementary material file. DOI: 10.1107/S1600536813021545/pv2642Isup3.cml

Additional supplementary materials:  crystallographic information; 3D view; checkCIF report

## Figures and Tables

**Table 1:** Hydrogen-bond geometry (Å, °) *Cg*1 is the centroid of the N1-pyridyl ring.

*D*—H⋯*A*	*D*—H	H⋯*A*	*D*⋯*A*	*D*—H⋯*A*
C10—H10*C*⋯O1^i^	0.98	2.54	3.4116 (18)	148
C15—H15⋯*Cg*1^ii^	0.95	2.65	3.4695 (15)	145

Symmetry codes: (i) 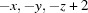; (ii) 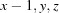.

**Fig. 1.**
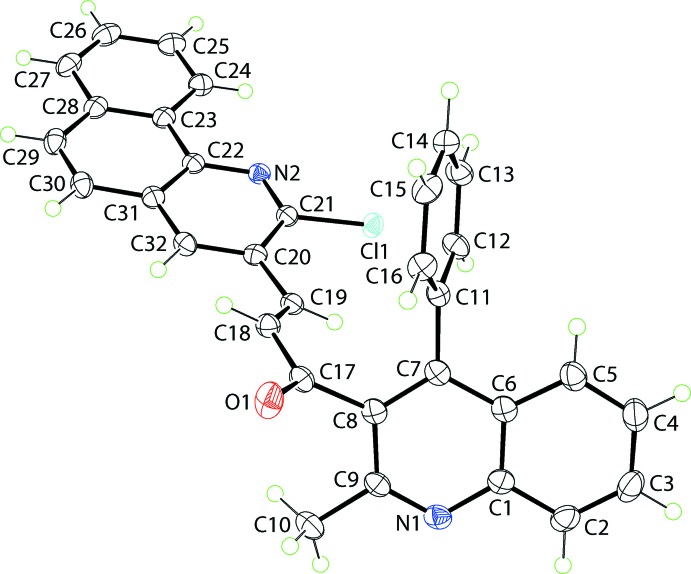
The molecular structure of (I) showing the atom-labelling scheme and displacement ellipsoids at the 70% probability level.

**Fig. 2.**
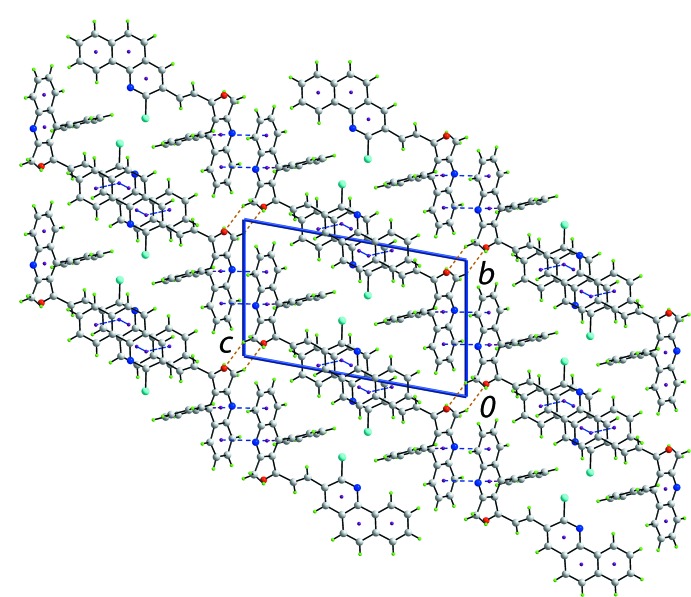
View in projection down the a axis of the unit-cell contents of (I). The C—H···O, C—H···π and π—π interactions are shown as orange, purple and blue dashed lines, respectively.

## supplementary crystallographic information

### 1. Comment

In connection with the biological potential of quinolinyl derivatives (Jörg *et al.* 2007; Prasath *et al.* 2013*a*), the title compound (I) was prepared and subjected to a crystallographic study.

The molecular structure of (I) (Fig. 1), sees two somewhat splayed [dihedral angle = 61.60 (3)°] quinolinyl and benzoquinolinyl residues connected by the ends of a planar (r.m.s. deviation = 0.033 Å) prop-2-en-1-one bridge. The quinolinyl, especially, and benzoquinolinyl residues are twisted out of the plane of the prop-2-en-1-one bridge forming dihedral angles of 75.94 (5) and 20.20 (5)°, respectively. The phenyl ring is inclined with respect to the quinolinyl residue to which it is attached, forming a dihedral angle of 73.76 (5)°. Finally, the conformation about the ethylene bond [C18═C19 = 1.3351 (18) Å] is *E*.

A similar conformation was reported recently for a related structure having two quinolinyl residues bridged by a prop-2-en-1-one residue, namely (*2E*)-3-(2-chloro-8-methylquinolin-3-yl)-1-(5,7-dimethylquinolin-6-yl)prop-2-en-1-one (Prasath *et al.*, 2013*b*) where the dihedral angle between the quinolinyl residues was 83.72 (4)°.

In the crystal packing methyl-*C*—*H*···*O* (carbonyl) interactions (Table 1), link molecules into centrosymmetric dimers which are connected into a three dimensional architecture by phenyl-C—H···π(N1-pyridyl) (Table 1), and π–π interactions [inter-centroid distances: *Cg*(N1-pyridyl)···*Cg*(C1–C6)^i^ = 3.7537 (7) Å, *Cg*(N2-pyridyl)···*Cg*(C22—C23,C28–C31)^ii,iii^ = 3.5806 (7) and 3.7286 (7) Å for *i* - *x*, 1 - *y*, 2 - *z*, *ii* 1 - *x*, -*y*, 1 - *z*, *iii* -*x*, -*y*, 1 - *z*] (Fig. 2).

### 2. Experimental

A mixture of 3-acetyl-2-methyl-4-phenylquinoline (260 mg, 0.001 *M*) and 2-chlorobenzoquinoline-3-carbaldehyde (240 mg, 0.001 *M*) in methanol (20 ml) containing potassium hydroxide (0.2 g) was stirred at room temperature for 12 h. After this, the reaction mixture was neutralized with dilute acetic acid and the resultant solid was filtered, dried and purified by column chromatography using an ethyl acetate - hexane (4:1) mixture to afford the title compound, (I). Re-crystallization was by slow evaporation of an acetone solution of (I), which yielded colourless blocks in 80% yield; *M*.pt: 460–462 K.

### 3. Refinement

Carbon-bound H-atoms were placed in calculated positions [C—H = 0.95–0.98 Å, *U*_iso_(H) = 1.2–1.5*U*_eq_(C)] and were included in the refinement in the riding model approximation.

### Crystal data

**Table:**

C_32_H_21_ClN_2_O	*Z* = 2
*M**_r_* = 484.96	*F*(000) = 504
Triclinic, *P*1	*D*_x_ = 1.357 Mg m^−^^3^
Hall symbol: -P 1	Cu *K*α radiation, λ = 1.54184 Å
*a* = 7.1354 (3) Å	Cell parameters from 5174 reflections
*b* = 10.1627 (5) Å	θ = 2.7–76.5°
*c* = 17.0127 (8) Å	µ = 1.65 mm^−^^1^
α = 78.758 (4)°	*T* = 100 K
β = 79.544 (4)°	Block, colourless
γ = 84.042 (4)°	0.30 × 0.25 × 0.20 mm
*V* = 1186.91 (9) Å^3^	

### Data collection

**Table:**

Agilent SuperNova Dual diffractometer with an Atlas detector	4849 independent reflections
Radiation source: SuperNova (Cu) X-ray Source	4433 reflections with *I* > 2σ(*I*)
Mirror monochromator	*R*_int_ = 0.020
Detector resolution: 10.4041 pixels mm^-1^	θ_max_ = 76.6°, θ_min_ = 2.7°
ω scan	*h* = −7→8
Absorption correction: multi-scan (*CrysAlis PRO*; Agilent, 2013)	*k* = −10→12
*T*_min_ = 0.870, *T*_max_ = 1.000	*l* = −15→21
8673 measured reflections	

### Refinement

**Table:**

Refinement on *F*^2^	Primary atom site location: structure-invariant direct methods
Least-squares matrix: full	Secondary atom site location: difference Fourier map
*R*[*F*^2^ > 2σ(*F*^2^)] = 0.034	Hydrogen site location: inferred from neighbouring sites
*wR*(*F*^2^) = 0.095	H-atom parameters constrained
*S* = 1.04	*w* = 1/[σ^2^(*F*_o_^2^) + (0.052*P*)^2^ + 0.2928*P*] where *P* = (*F*_o_^2^ + 2*F*_c_^2^)/3
4849 reflections	(Δ/σ)_max_ = 0.002
326 parameters	Δρ_max_ = 0.24 e Å^−^^3^
0 restraints	Δρ_min_ = −0.31 e Å^−^^3^

### Special details

**Table:**

Geometry. All s.u.'s (except the s.u. in the dihedral angle between two l.s. planes) are estimated using the full covariance matrix. The cell s.u.'s are taken into account individually in the estimation of s.u.'s in distances, angles and torsion angles; correlations between s.u.'s in cell parameters are only used when they are defined by crystal symmetry. An approximate (isotropic) treatment of cell s.u.'s is used for estimating s.u.'s involving l.s. planes.
Refinement. Refinement of *F*^2^ against ALL reflections. The weighted *R*-factor *wR* and goodness of fit *S* are based on *F*^2^, conventional *R*-factors *R* are based on *F*, with *F* set to zero for negative *F*^2^. The threshold expression of *F*^2^ > 2σ(*F*^2^) is used only for calculating *R*-factors(gt) *etc*. and is not relevant to the choice of reflections for refinement. *R*-factors based on *F*^2^ are statistically about twice as large as those based on *F*, and *R*- factors based on ALL data will be even larger.

### Fractional atomic coordinates and isotropic or equivalent isotropic displacement parameters (Å^2^)

**Table:**

	*x*	*y*	*z*	*U*_iso_*/*U*_eq_	
Cl1	0.32862 (4)	0.39004 (3)	0.557615 (17)	0.02330 (9)	
O1	−0.10222 (16)	0.11751 (11)	0.91090 (6)	0.0351 (3)	
N1	0.29529 (16)	0.38332 (11)	0.94697 (6)	0.0245 (2)	
N2	0.31428 (15)	0.18592 (10)	0.48984 (6)	0.0194 (2)	
C1	0.22372 (19)	0.51192 (13)	0.92151 (7)	0.0228 (3)	
C2	0.2945 (2)	0.61806 (15)	0.94820 (8)	0.0295 (3)	
H2	0.3912	0.5989	0.9814	0.035*	
C3	0.2242 (2)	0.74768 (15)	0.92635 (9)	0.0348 (3)	
H3	0.2719	0.8182	0.9447	0.042*	
C4	0.0813 (2)	0.77789 (15)	0.87687 (9)	0.0346 (3)	
H4	0.0311	0.8682	0.8634	0.042*	
C5	0.0143 (2)	0.67797 (14)	0.84801 (8)	0.0283 (3)	
H5	−0.0798	0.6997	0.8136	0.034*	
C6	0.08475 (18)	0.54237 (13)	0.86931 (7)	0.0217 (3)	
C7	0.01900 (18)	0.43308 (13)	0.84298 (7)	0.0204 (2)	
C8	0.08610 (18)	0.30408 (13)	0.87255 (7)	0.0208 (3)	
C9	0.22715 (18)	0.28343 (13)	0.92474 (7)	0.0225 (3)	
C10	0.3081 (2)	0.14360 (14)	0.95496 (9)	0.0308 (3)	
H10A	0.4311	0.1487	0.9718	0.046*	
H10B	0.3267	0.0908	0.9114	0.046*	
H10C	0.2192	0.1004	1.0014	0.046*	
C11	−0.11840 (18)	0.46154 (12)	0.78448 (8)	0.0210 (2)	
C12	−0.05441 (19)	0.51971 (13)	0.70407 (8)	0.0246 (3)	
H12	0.0762	0.5378	0.6873	0.029*	
C13	−0.1793 (2)	0.55154 (14)	0.64831 (8)	0.0262 (3)	
H13	−0.1337	0.5896	0.5934	0.031*	
C14	−0.3710 (2)	0.52756 (13)	0.67300 (8)	0.0259 (3)	
H14	−0.4576	0.5513	0.6353	0.031*	
C15	−0.43578 (19)	0.46909 (14)	0.75253 (9)	0.0273 (3)	
H15	−0.5668	0.4523	0.7693	0.033*	
C16	−0.30955 (19)	0.43462 (14)	0.80842 (8)	0.0250 (3)	
H16	−0.3543	0.3929	0.8627	0.030*	
C17	0.00319 (19)	0.18359 (13)	0.85606 (8)	0.0224 (3)	
C18	0.04774 (18)	0.14195 (13)	0.77665 (8)	0.0217 (3)	
H18	−0.0178	0.0691	0.7701	0.026*	
C19	0.17108 (18)	0.19583 (12)	0.71255 (8)	0.0202 (2)	
H19	0.2367	0.2702	0.7168	0.024*	
C20	0.20927 (17)	0.14471 (13)	0.63586 (7)	0.0194 (2)	
C21	0.28047 (17)	0.22343 (12)	0.56031 (8)	0.0190 (2)	
C22	0.27867 (17)	0.05826 (12)	0.48717 (7)	0.0190 (2)	
C23	0.31445 (17)	0.01556 (13)	0.40911 (8)	0.0211 (3)	
C24	0.38163 (19)	0.10284 (14)	0.33679 (8)	0.0246 (3)	
H24	0.4041	0.1927	0.3385	0.030*	
C25	0.4152 (2)	0.05881 (15)	0.26338 (9)	0.0288 (3)	
H25	0.4604	0.1183	0.2148	0.035*	
C26	0.3824 (2)	−0.07406 (16)	0.26052 (9)	0.0297 (3)	
H26	0.4060	−0.1041	0.2099	0.036*	
C27	0.31638 (19)	−0.16072 (15)	0.33042 (9)	0.0279 (3)	
H27	0.2949	−0.2503	0.3277	0.033*	
C28	0.28016 (18)	−0.11821 (13)	0.40624 (8)	0.0230 (3)	
C29	0.20946 (19)	−0.20770 (13)	0.47966 (9)	0.0256 (3)	
H29	0.1870	−0.2972	0.4772	0.031*	
C30	0.17442 (18)	−0.16655 (13)	0.55216 (9)	0.0243 (3)	
H30	0.1267	−0.2272	0.5998	0.029*	
C31	0.20836 (17)	−0.03237 (13)	0.55800 (8)	0.0209 (2)	
C32	0.17548 (17)	0.01381 (13)	0.63202 (8)	0.0208 (2)	
H32	0.1292	−0.0454	0.6806	0.025*	

### Atomic displacement parameters (Å^2^)

**Table:**

	*U*^11^	*U*^22^	*U*^33^	*U*^12^	*U*^13^	*U*^23^
Cl1	0.03021 (17)	0.01941 (15)	0.02004 (15)	−0.00805 (11)	−0.00196 (11)	−0.00167 (11)
O1	0.0475 (6)	0.0300 (5)	0.0246 (5)	−0.0175 (5)	0.0085 (4)	−0.0029 (4)
N1	0.0274 (6)	0.0271 (6)	0.0169 (5)	−0.0041 (4)	−0.0017 (4)	0.0003 (4)
N2	0.0178 (5)	0.0207 (5)	0.0196 (5)	−0.0021 (4)	−0.0038 (4)	−0.0022 (4)
C1	0.0258 (6)	0.0256 (6)	0.0150 (6)	−0.0046 (5)	0.0015 (5)	−0.0015 (5)
C2	0.0380 (8)	0.0324 (7)	0.0184 (6)	−0.0089 (6)	−0.0022 (5)	−0.0045 (5)
C3	0.0529 (10)	0.0289 (7)	0.0241 (7)	−0.0119 (7)	−0.0008 (6)	−0.0091 (6)
C4	0.0515 (10)	0.0225 (7)	0.0276 (7)	0.0001 (6)	−0.0015 (6)	−0.0050 (5)
C5	0.0343 (7)	0.0245 (7)	0.0234 (6)	0.0005 (5)	−0.0012 (5)	−0.0028 (5)
C6	0.0232 (6)	0.0226 (6)	0.0160 (6)	−0.0029 (5)	0.0032 (5)	−0.0010 (5)
C7	0.0196 (6)	0.0223 (6)	0.0162 (5)	−0.0027 (5)	0.0026 (4)	−0.0004 (4)
C8	0.0215 (6)	0.0222 (6)	0.0160 (6)	−0.0038 (5)	0.0024 (4)	−0.0005 (4)
C9	0.0239 (6)	0.0232 (6)	0.0170 (6)	−0.0031 (5)	−0.0001 (5)	0.0021 (5)
C10	0.0349 (8)	0.0256 (7)	0.0288 (7)	−0.0013 (6)	−0.0074 (6)	0.0041 (5)
C11	0.0222 (6)	0.0186 (6)	0.0209 (6)	−0.0011 (5)	−0.0019 (5)	−0.0019 (5)
C12	0.0224 (6)	0.0242 (6)	0.0237 (6)	−0.0028 (5)	−0.0021 (5)	0.0025 (5)
C13	0.0299 (7)	0.0252 (6)	0.0215 (6)	−0.0033 (5)	−0.0048 (5)	0.0017 (5)
C14	0.0275 (7)	0.0239 (6)	0.0281 (7)	0.0002 (5)	−0.0097 (5)	−0.0058 (5)
C15	0.0211 (6)	0.0328 (7)	0.0296 (7)	−0.0053 (5)	−0.0012 (5)	−0.0099 (6)
C16	0.0240 (7)	0.0290 (7)	0.0211 (6)	−0.0045 (5)	0.0003 (5)	−0.0047 (5)
C17	0.0239 (6)	0.0205 (6)	0.0200 (6)	−0.0029 (5)	−0.0009 (5)	0.0014 (5)
C18	0.0217 (6)	0.0216 (6)	0.0212 (6)	−0.0039 (5)	−0.0039 (5)	−0.0010 (5)
C19	0.0204 (6)	0.0198 (6)	0.0199 (6)	−0.0023 (5)	−0.0051 (5)	−0.0003 (5)
C20	0.0152 (6)	0.0227 (6)	0.0200 (6)	−0.0024 (4)	−0.0032 (4)	−0.0021 (5)
C21	0.0165 (6)	0.0192 (6)	0.0212 (6)	−0.0031 (4)	−0.0041 (4)	−0.0012 (5)
C22	0.0141 (5)	0.0215 (6)	0.0214 (6)	−0.0013 (4)	−0.0039 (4)	−0.0027 (5)
C23	0.0153 (6)	0.0240 (6)	0.0250 (6)	0.0002 (5)	−0.0049 (5)	−0.0059 (5)
C24	0.0230 (6)	0.0274 (6)	0.0238 (6)	−0.0003 (5)	−0.0046 (5)	−0.0055 (5)
C25	0.0259 (7)	0.0367 (8)	0.0244 (7)	−0.0003 (6)	−0.0047 (5)	−0.0073 (6)
C26	0.0239 (7)	0.0413 (8)	0.0276 (7)	0.0011 (6)	−0.0055 (5)	−0.0157 (6)
C27	0.0214 (6)	0.0316 (7)	0.0350 (7)	0.0007 (5)	−0.0081 (5)	−0.0150 (6)
C28	0.0153 (6)	0.0261 (6)	0.0293 (7)	0.0001 (5)	−0.0053 (5)	−0.0086 (5)
C29	0.0201 (6)	0.0218 (6)	0.0363 (7)	−0.0024 (5)	−0.0054 (5)	−0.0073 (5)
C30	0.0205 (6)	0.0210 (6)	0.0304 (7)	−0.0037 (5)	−0.0025 (5)	−0.0023 (5)
C31	0.0162 (6)	0.0215 (6)	0.0246 (6)	−0.0014 (5)	−0.0039 (5)	−0.0029 (5)
C32	0.0179 (6)	0.0207 (6)	0.0217 (6)	−0.0038 (5)	−0.0014 (5)	0.0007 (5)

### Geometric parameters (Å, º)

**Table:**

Cl1—C21	1.7526 (12)	C14—C15	1.383 (2)
O1—C17	1.2219 (16)	C14—H14	0.9500
N1—C9	1.3149 (17)	C15—C16	1.3976 (19)
N1—C1	1.3703 (17)	C15—H15	0.9500
N2—C21	1.3019 (16)	C16—H16	0.9500
N2—C22	1.3585 (16)	C17—C18	1.4643 (18)
C1—C2	1.4187 (19)	C18—C19	1.3351 (18)
C1—C6	1.4200 (18)	C18—H18	0.9500
C2—C3	1.366 (2)	C19—C20	1.4671 (17)
C2—H2	0.9500	C19—H19	0.9500
C3—C4	1.410 (2)	C20—C32	1.3923 (17)
C3—H3	0.9500	C20—C21	1.4130 (17)
C4—C5	1.372 (2)	C22—C31	1.4127 (18)
C4—H4	0.9500	C22—C23	1.4477 (17)
C5—C6	1.4180 (18)	C23—C24	1.4067 (19)
C5—H5	0.9500	C23—C28	1.4176 (18)
C6—C7	1.4257 (18)	C24—C25	1.3801 (19)
C7—C8	1.3788 (18)	C24—H24	0.9500
C7—C11	1.4895 (18)	C25—C26	1.406 (2)
C8—C9	1.4328 (18)	C25—H25	0.9500
C8—C17	1.5075 (17)	C26—C27	1.373 (2)
C9—C10	1.5067 (18)	C26—H26	0.9500
C10—H10A	0.9800	C27—C28	1.4108 (19)
C10—H10B	0.9800	C27—H27	0.9500
C10—H10C	0.9800	C28—C29	1.437 (2)
C11—C16	1.3905 (18)	C29—C30	1.351 (2)
C11—C12	1.3940 (18)	C29—H29	0.9500
C12—C13	1.3877 (19)	C30—C31	1.4347 (17)
C12—H12	0.9500	C30—H30	0.9500
C13—C14	1.389 (2)	C31—C32	1.4005 (18)
C13—H13	0.9500	C32—H32	0.9500
			
C9—N1—C1	118.65 (12)	C11—C16—H16	120.1
C21—N2—C22	117.81 (11)	C15—C16—H16	120.1
N1—C1—C2	117.88 (12)	O1—C17—C18	118.97 (12)
N1—C1—C6	122.75 (12)	O1—C17—C8	119.05 (12)
C2—C1—C6	119.37 (12)	C18—C17—C8	121.98 (11)
C3—C2—C1	120.19 (14)	C19—C18—C17	126.68 (12)
C3—C2—H2	119.9	C19—C18—H18	116.7
C1—C2—H2	119.9	C17—C18—H18	116.7
C2—C3—C4	120.63 (13)	C18—C19—C20	122.60 (11)
C2—C3—H3	119.7	C18—C19—H19	118.7
C4—C3—H3	119.7	C20—C19—H19	118.7
C5—C4—C3	120.53 (14)	C32—C20—C21	114.63 (11)
C5—C4—H4	119.7	C32—C20—C19	122.08 (11)
C3—C4—H4	119.7	C21—C20—C19	123.29 (11)
C4—C5—C6	120.27 (14)	N2—C21—C20	126.73 (11)
C4—C5—H5	119.9	N2—C21—Cl1	114.54 (9)
C6—C5—H5	119.9	C20—C21—Cl1	118.72 (9)
C5—C6—C1	118.95 (12)	N2—C22—C31	121.81 (11)
C5—C6—C7	123.36 (12)	N2—C22—C23	118.37 (11)
C1—C6—C7	117.66 (12)	C31—C22—C23	119.83 (11)
C8—C7—C6	118.52 (12)	C24—C23—C28	119.61 (12)
C8—C7—C11	122.21 (11)	C24—C23—C22	121.87 (12)
C6—C7—C11	119.26 (11)	C28—C23—C22	118.53 (12)
C7—C8—C9	119.66 (12)	C25—C24—C23	120.44 (13)
C7—C8—C17	121.10 (12)	C25—C24—H24	119.8
C9—C8—C17	119.07 (11)	C23—C24—H24	119.8
N1—C9—C8	122.61 (12)	C24—C25—C26	120.01 (14)
N1—C9—C10	116.89 (12)	C24—C25—H25	120.0
C8—C9—C10	120.48 (12)	C26—C25—H25	120.0
C9—C10—H10A	109.5	C27—C26—C25	120.43 (13)
C9—C10—H10B	109.5	C27—C26—H26	119.8
H10A—C10—H10B	109.5	C25—C26—H26	119.8
C9—C10—H10C	109.5	C26—C27—C28	120.74 (13)
H10A—C10—H10C	109.5	C26—C27—H27	119.6
H10B—C10—H10C	109.5	C28—C27—H27	119.6
C16—C11—C12	119.22 (12)	C27—C28—C23	118.77 (13)
C16—C11—C7	121.74 (11)	C27—C28—C29	121.20 (12)
C12—C11—C7	119.03 (11)	C23—C28—C29	120.03 (12)
C13—C12—C11	120.78 (12)	C30—C29—C28	121.05 (12)
C13—C12—H12	119.6	C30—C29—H29	119.5
C11—C12—H12	119.6	C28—C29—H29	119.5
C12—C13—C14	119.79 (13)	C29—C30—C31	120.81 (13)
C12—C13—H13	120.1	C29—C30—H30	119.6
C14—C13—H13	120.1	C31—C30—H30	119.6
C15—C14—C13	119.88 (13)	C32—C31—C22	117.68 (11)
C15—C14—H14	120.1	C32—C31—C30	122.58 (12)
C13—C14—H14	120.1	C22—C31—C30	119.74 (12)
C14—C15—C16	120.41 (12)	C20—C32—C31	121.34 (12)
C14—C15—H15	119.8	C20—C32—H32	119.3
C16—C15—H15	119.8	C31—C32—H32	119.3
C11—C16—C15	119.90 (12)		
			
C9—N1—C1—C2	178.03 (12)	C9—C8—C17—C18	−109.35 (14)
C9—N1—C1—C6	−2.71 (18)	O1—C17—C18—C19	−174.33 (14)
N1—C1—C2—C3	−178.22 (12)	C8—C17—C18—C19	4.6 (2)
C6—C1—C2—C3	2.5 (2)	C17—C18—C19—C20	178.53 (12)
C1—C2—C3—C4	−0.3 (2)	C18—C19—C20—C32	−22.79 (19)
C2—C3—C4—C5	−1.8 (2)	C18—C19—C20—C21	156.45 (12)
C3—C4—C5—C6	1.5 (2)	C22—N2—C21—C20	−0.07 (19)
C4—C5—C6—C1	0.7 (2)	C22—N2—C21—Cl1	−179.46 (9)
C4—C5—C6—C7	178.69 (13)	C32—C20—C21—N2	0.69 (19)
N1—C1—C6—C5	178.07 (12)	C19—C20—C21—N2	−178.60 (11)
C2—C1—C6—C5	−2.69 (18)	C32—C20—C21—Cl1	−179.95 (9)
N1—C1—C6—C7	−0.04 (18)	C19—C20—C21—Cl1	0.76 (16)
C2—C1—C6—C7	179.20 (12)	C21—N2—C22—C31	−0.37 (17)
C5—C6—C7—C8	−174.86 (12)	C21—N2—C22—C23	179.59 (11)
C1—C6—C7—C8	3.16 (17)	N2—C22—C23—C24	−0.97 (18)
C5—C6—C7—C11	5.43 (19)	C31—C22—C23—C24	179.00 (11)
C1—C6—C7—C11	−176.55 (11)	N2—C22—C23—C28	179.08 (11)
C6—C7—C8—C9	−3.58 (18)	C31—C22—C23—C28	−0.95 (17)
C11—C7—C8—C9	176.12 (11)	C28—C23—C24—C25	−0.35 (19)
C6—C7—C8—C17	171.82 (11)	C22—C23—C24—C25	179.70 (12)
C11—C7—C8—C17	−8.49 (18)	C23—C24—C25—C26	−0.1 (2)
C1—N1—C9—C8	2.33 (19)	C24—C25—C26—C27	0.2 (2)
C1—N1—C9—C10	−179.25 (11)	C25—C26—C27—C28	0.1 (2)
C7—C8—C9—N1	0.84 (19)	C26—C27—C28—C23	−0.46 (19)
C17—C8—C9—N1	−174.65 (11)	C26—C27—C28—C29	179.43 (12)
C7—C8—C9—C10	−177.52 (12)	C24—C23—C28—C27	0.59 (18)
C17—C8—C9—C10	6.99 (18)	C22—C23—C28—C27	−179.45 (11)
C8—C7—C11—C16	73.14 (17)	C24—C23—C28—C29	−179.29 (12)
C6—C7—C11—C16	−107.17 (14)	C22—C23—C28—C29	0.66 (18)
C8—C7—C11—C12	−108.44 (14)	C27—C28—C29—C30	−179.82 (12)
C6—C7—C11—C12	71.26 (16)	C23—C28—C29—C30	0.07 (19)
C16—C11—C12—C13	0.4 (2)	C28—C29—C30—C31	−0.5 (2)
C7—C11—C12—C13	−178.03 (12)	N2—C22—C31—C32	0.16 (18)
C11—C12—C13—C14	1.2 (2)	C23—C22—C31—C32	−179.81 (11)
C12—C13—C14—C15	−1.6 (2)	N2—C22—C31—C30	−179.50 (11)
C13—C14—C15—C16	0.4 (2)	C23—C22—C31—C30	0.53 (18)
C12—C11—C16—C15	−1.64 (19)	C29—C30—C31—C32	−179.43 (12)
C7—C11—C16—C15	176.78 (12)	C29—C30—C31—C22	0.21 (19)
C14—C15—C16—C11	1.3 (2)	C21—C20—C32—C31	−0.87 (18)
C7—C8—C17—O1	−105.83 (15)	C19—C20—C32—C31	178.43 (11)
C9—C8—C17—O1	69.59 (17)	C22—C31—C32—C20	0.50 (18)
C7—C8—C17—C18	75.23 (16)	C30—C31—C32—C20	−179.85 (12)

### Hydrogen-bond geometry (Å, º)

Cg1 is the centroid of the N1-pyridyl ring.

**Table:**

*D*—H···*A*	*D*—H	H···*A*	*D*···*A*	*D*—H···*A*
C10—H10*C*···O1^i^	0.98	2.54	3.4116 (18)	148
C15—H15···*Cg*1^ii^	0.95	2.65	3.4695 (15)	145

Symmetry codes: (i) −*x*, −*y*, −*z*+2; (ii) *x*−1, *y*, *z*.

## References

[bb1] Agilent (2013). *CrysAlis PRO* Agilent Technologies Inc., Santa Clara, CA, USA.

[bb2] Brandenburg, K. (2006). *DIAMOND* Crystal Impact GbR, Bonn, Germany.

[bb3] Farrugia, L. J. (2012). *J. Appl. Cryst.* **45**, 849–854.

[bb4] Jörg, W., Daniela, G. & Gerhard, R. (2007). *Inorg. Chim. Acta*, **360**, 1935–1942.

[bb5] Prasath, R., Bhavana, P., Ng, S. W. & Tiekink, E. R. T. (2013*a*). *J. Organomet. Chem.* **726**, 62–70.

[bb6] Prasath, R., Sarveswari, S., Ng, S. W. & Tiekink, E. R. T. (2013*b*). *Acta Cryst.* E**69**, o1275. 10.1107/S1600536813019405 PMC3793770 24109357

[bb7] Sheldrick, G. M. (2008). *Acta Cryst.* A**64**, 112–122. 10.1107/S0108767307043930 18156677

[bb8] Westrip, S. P. (2010). *J. Appl. Cryst.* **43**, 920–925.

